# Supplementary material for: Optimizing testing for COVID-19 in India
Source: PLoS Comput Biol. 2021 Jul 22;17(7):e1009126. doi: 10.1371/journal.pcbi.1009126 (PMC8297905; doi:10.1371/journal.pcbi.1009126)
Supplement: S1 Appendix — The dynamics of the disease-progression in a single well-mixed compartmental model are studied, and the reproductive ratio is calculated by applying a next-generation matrix method to the governing equations. The result is that for a single well-mixed compartment, the reproductive ratio R0 is R0=λS(γλA+(1-γ)(1λP+δλMI+(1-δ)(1λSI+σλH))). Using the parameters given in Table 1, this yields R0 = 2.374. (PDF) [file pcbi.1009126.s001.pdf]

S1 Appendix: Analysis of Compartmental Model

As mentioned in the main text, our model contains 7-compartments. These are (S)usceptible, (A)symptomatic, (P)resymptomatic, Mildly Infected (MI), Severely Infected (SI), (H)ospitalized, and (R)ecovered. These are shown in Fig 1 of the main text. We ignore deaths, although they could be easily incorporated into this general framework [1].

Coupled ODEs for the compartmental model for a single well-mixed location

If we assume the population of N individuals to be in a single well-mixed location, the dynamics of our compartmental model can be represented by the following equations [1, 2]:

$$\frac{\mathrm{d}S}{\mathrm{d}t} = -\frac{\lambda_S}{N} S(A + P + MI + SI + H), \tag{1}$$

$$\frac{\mathrm{d}A}{\mathrm{d}t} = \gamma \frac{\lambda_S}{N} S(A + P + MI + SI + H) - \lambda_A A, \tag{2}$$

$$\frac{\mathrm{d}P}{\mathrm{d}t} = (1 - \gamma) \frac{\lambda_S}{N} S(A + P + MI + SI + H) - \lambda_P P, \tag{3}$$

$$\frac{\mathrm{d}MI}{\mathrm{d}t} = \delta \lambda_P P - \lambda_{MI} MI, \tag{4}$$

$$\frac{\mathrm{d}SI}{\mathrm{d}t} = (1 - \delta) \lambda_P P - \lambda_{SI} SI, \tag{5}$$

$$\frac{\mathrm{d}H}{\mathrm{d}t} = \sigma \lambda_{SI} SI - \lambda_H H, \tag{6}$$

$$\frac{\mathrm{d}R}{\mathrm{d}t} = \lambda_A A + \lambda_{MI} MI + (1 - \sigma) \lambda_{SI} SI + \lambda_H H, \tag{7}$$

where the total number of individuals, $N \equiv S+A+P+MI+SI+H+R$ is constant. As mentioned in the main text, we use the parameters given in Table 1, except when explicitly stated.

Reproduction Number: Next Generation Matrix Method

A next-generation matrix method applied to the equations above allows us to calculate the basic reproductive ratio [3]. We start off by rearranging the equations into two different compartments, infected and non-infected:

$$X = \begin{pmatrix} A \\ P \\ MI \\ SI \\ H \end{pmatrix}, \qquad Y = \begin{pmatrix} S \\ R \end{pmatrix}$$

Defining two new vectors $\mathcal{F}$ and $\mathcal{V}$ such that

$$\frac{\mathrm{d}X}{\mathrm{d}t} = (\mathcal{F} - \mathcal{V}),$$

where $\mathcal{F}$ is the vector of **new** infection rates (flows from Y to X), and $\mathcal{V}$ is the vector of all **other** rates (no new infections), which include flows from X to Y, and flows within X. For each compartment, the inflow terms in $\mathcal{V}$ are negative, and the outflow is positive.

From these vectors, we can define two matrices evaluated at the Disease Free Equilibrium (DFE)

$$F = \left(\frac{\partial \mathcal{F}}{\partial X}\right)\bigg|_{\text{At DFE}} \qquad V = \left(\frac{\partial \mathcal{V}}{\partial X}\right)\bigg|_{\text{At DFE}},$$

such that the next generation matrix is $G = FV^{-1}$. The reproduction number R_0 is the spectral radius (i.e. maximum eigenvalue) of G, i.e. $R_0 = \rho(FV^{-1})$.

Note: The Disease Free Equilibrium occurs when $I = A + P + MI + SI + H = 0$, i.e. when $\dot{S} = 0$, or $S = N$.

$$\mathcal{F} = \begin{pmatrix} \gamma \frac{\lambda_S}{N} S(A+P+MI+SI+H) \\ (1-\gamma)\frac{\lambda_S}{N} S(A+P+MI+SI+H) \\ 0 \\ 0 \\ 0 \end{pmatrix}$$

$$\implies F = \frac{\lambda_S}{N} S^* \begin{pmatrix} \gamma & \gamma & \gamma & \gamma & \gamma \\ (1-\gamma) & (1-\gamma) & (1-\gamma) & (1-\gamma) & (1-\gamma) \\ 0 & 0 & 0 & 0 & 0 \\ 0 & 0 & 0 & 0 & 0 \\ 0 & 0 & 0 & 0 & 0 \end{pmatrix}$$

$$\mathcal{V} = \begin{pmatrix} \lambda_A A \\ \lambda_P P \\ \lambda_{MI} MI - \delta\lambda_P P \\ \lambda_{SI} SI - (1-\delta)\lambda_P P \\ \lambda_H H - \sigma\lambda_{SI} SI \end{pmatrix} \implies V = \begin{pmatrix} \lambda_A & 0 & 0 & 0 & 0 \\ 0 & \lambda_P & 0 & 0 & 0 \\ 0 & -\delta\lambda_P & \lambda_{MI} & 0 & 0 \\ 0 & -(1-\delta)\lambda_P & 0 & \lambda_{SI} & 0 \\ 0 & 0 & 0 & -\sigma\lambda_{SI} & \lambda_H \end{pmatrix}$$

Using this we can compute the next-generation matrix,

$$G = FV^{-1} =$$

$$\begin{pmatrix} \frac{\gamma\lambda_S}{\lambda_A} & \gamma\lambda_S\left(\frac{1}{\lambda_P} + \frac{\delta}{\lambda_{MI}} + (1-\delta)\left(\frac{1}{\lambda_{SI}} + \frac{\sigma}{\lambda_H}\right)\right) & \frac{\gamma\lambda_S}{\lambda_{MI}} & \gamma\lambda_S\left(\frac{1}{\lambda_{SI}} + \frac{\sigma}{\lambda_H}\right) & \gamma\frac{\lambda_S}{\lambda_H} \\ \frac{(1-\gamma)\lambda_S}{\lambda_A} & (1-\gamma)\lambda_S\left(\frac{1}{\lambda_P} + \frac{\delta}{\lambda_{MI}} + (1-\delta)\left(\frac{1}{\lambda_{SI}} + \frac{\sigma}{\lambda_H}\right)\right) & \frac{(1-\gamma)\lambda_S}{\lambda_{MI}} & (1-\gamma)\lambda_S\left(\frac{1}{\lambda_{SI}} + \frac{\sigma}{\lambda_H}\right) & \frac{(1-\gamma)\lambda_S}{\lambda_H} \\ 0 & 0 & 0 & 0 & 0 \\ 0 & 0 & 0 & 0 & 0 \\ 0 & 0 & 0 & 0 & 0 \end{pmatrix}$$

The eigenvalues are $\left\{0, 0, 0, 0, \lambda_S\left(\frac{\gamma}{\lambda_A} + (1-\gamma)\left(\frac{1}{\lambda_P} + \frac{\delta}{\lambda_{MI}} + (1-\delta)\left(\frac{1}{\lambda_{SI}} + \frac{\sigma}{\lambda_H}\right)\right)\right)\right\}$. Thus

$$R_0 = \lambda_S\left(\frac{\gamma}{\lambda_A} + (1-\gamma)\left(\frac{1}{\lambda_P} + \frac{\delta}{\lambda_{MI}} + (1-\delta)\left(\frac{1}{\lambda_{SI}} + \frac{\sigma}{\lambda_H}\right)\right)\right).$$

Using the default parameter values given in Table 1 in the main text:

$$R_0 = 2.374.$$

References

1. Keeling MJ, Rohani P. Modeling Infectious Diseases in Humans and Animals. Illustrated edition ed. Princeton: Princeton University Press; 2007.

2. Anderson RM, May RM, Anderson B. Infectious Diseases of Humans: Dynamics and Control. Revised ed. edition ed. Oxford: Oxford University Press; 1992.

3. Diekmann O, Heesterbeek JaP, Roberts MG. The construction of next-generation matrices for compartmental epidemic models. Journal of The Royal Society Interface. 2010;7(47):873–885. doi:10.1098/rsif.2009.0386.
