# Supplementary material for: Optimizing testing for COVID-19 in India
Source: PLoS Comput Biol. 2021 Jul 22;17(7):e1009126. doi: 10.1371/journal.pcbi.1009126 (PMC8297905; doi:10.1371/journal.pcbi.1009126)
Supplement: S2 Appendix — The results of the main text are obtained for a population size of 10,000 individuals, with 2,750 locations (2,500 home locations and 250 work locations). This corresponds to a Poisson distributed population in the home and work locations with a mean of 4 and 40 individuals respectively. We also started with an initial infection seed of 10 asymptomatic individuals (0.1% of the population). However, we also tried a number of runs that were scaled-up to larger population sizes (while keeping the initial infection fraction the same, and preserving household and work location sizes) in order to check for finite-size effects. The results of these simulations are shown in the figure, and they are found to agree remarkably well with our results for 10,000 individuals, indicating that this problem scales well with the total population. (PDF) [file pcbi.1009126.s002.pdf]

S2 Appendix: Scaling up our simulation

The results of the main text are obtained for a population size of 10,000 individuals, with 2,750 locations (2,500 home locations and 250 work locations). This corresponds to a Poisson distributed population in the home and work locations with a mean of 4 and 40 individuals respectively. We also started with an initial infection seed of 10 asymptomatic individuals (0.1% of the population). However, we also tried a number of runs that were scaled-up to larger population sizes (while keeping the initial infection fraction the same, and preserving household and work location sizes) in order to check for finite-size effects. The results of these simulations are shown in S2.1 Fig, and they are found to agree remarkably well with our results for 10,000 individuals, indicating that this problem scales well with the total population.

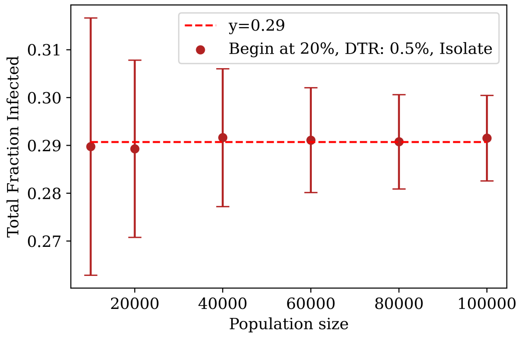

(a) Variation with population size. (DTR = 0.5%, starting at 20% recovered, only PCR tests)

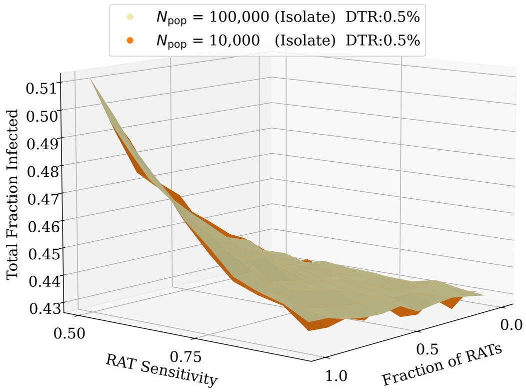

(b) Stacks for a DTR 0.5%, starting at 10% recovered with $N_{\text{pop}} = 10,000$ and $100,000$.

S2.1 Fig: Effects of scaling up the population. (a) Runs were conducted for different population sizes up to 100,000, with a daily testing rate of 0.5% and testing starting when 20% of the population had recovered. The results of the total infected fraction are shown for each of these populations, with the horizontal line representing their least-square fit. (b) A stack similar to those shown in the main text was also produced to compare the results for population sizes of 10,000 and 100,000, with a daily testing rate of 0.5% and with testing starting at 10%. The results show agreement over the range of RAT test sensitivities and fractions in the mixture. In both the above cases, the individuals who tested positive were isolated when they were declared.
