# Supplementary material for: Optimizing testing for COVID-19 in India
Source: PLoS Comput Biol. 2021 Jul 22;17(7):e1009126. doi: 10.1371/journal.pcbi.1009126 (PMC8297905; doi:10.1371/journal.pcbi.1009126)
Supplement: S3 Appendix — For random testing, available tests are distributed among eligible individuals in the population completely randomly, with no preference given to symptomatic individuals. The figure shows the effects of purely random testing, demonstrating the importance of first targeting symptomatics to reduce the total number of infections over the course of the pandemic. (PDF) [file pcbi.1009126.s003.pdf]

S3 Appendix: The effects of Random Testing

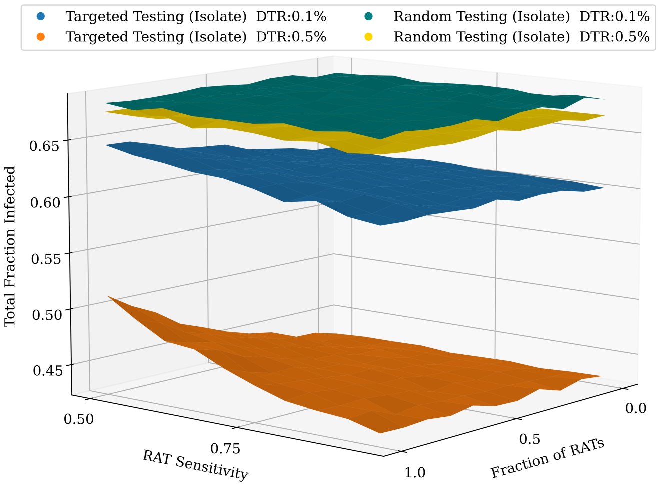

S3.1 Fig: Effects of random testing. If the testing is assumed to be purely random, with individuals picked out randomly from the population with no preference given to symptomatic individuals, the efficiency of testing is extremely low, as can be seen. While an increase in the daily testing does reduce the total infected fraction at the end of the pandemic, it is nowhere near as effective as targeted testing at the testing rates employed, demonstrating that it is extremely desirable to target symptomatics.
