# Supplementary material for: Optimizing testing for COVID-19 in India
Source: PLoS Comput Biol. 2021 Jul 22;17(7):e1009126. doi: 10.1371/journal.pcbi.1009126 (PMC8297905; doi:10.1371/journal.pcbi.1009126)
Supplement: S4 Appendix — As shown in the main text, the benefit of the PCR tests’ sensitivity is offset by the introduction of the delay. However, this can be countered by quarantining individuals or homes when the samples are taken. When large fractions of RAT tests are used (RAT:PCR ∼ 80:20) equivalent or marginally better results can be obtained by quarantining the homes of the tested individual when they are declared positive, instead of isolating them when they are sampled. (PDF) [file pcbi.1009126.s004.pdf]

# S4 Appendix: Effects of test delays on quarantining strategies

When no test delays are included, the results of tests are obtained immediately. There are thus only two different quarantining strategies possible:

(a) **Isolate:** Isolate the individual at home, reducing their infectivity and restricting their movement for 14 days,

(b) **Isolate and quarantine:** In addition to the above, also quarantine the entire home by restricting movement of all family members.

However, with the addition of a test delay, we can now make a distinction between enforcing the intervention when the individual is *sampled* and enforcing it when the result is *declared*. As a result, four different quarantining strategies are now possible:

(a) **Isolate when declared:** Isolate the individual at home, reducing their infectivity and restricting their movement for 14 days when the individual tests positive,

(b) **Isolate and quarantine when declared:** In addition to the above, also quarantine the entire home by restricting movement of all family members when the individual tests positive.

(c) **Isolate when sampled:** Isolate the individual at home, reducing their infectivity and restricting their movement when the individual is sampled for a test. If the test result is negative, the individual is released from isolation. If it is positive, they are further isolated for 14 days.

(d) **Isolate and quarantine when sampled:** In addition to the previous point, the individual's home is also quarantined until the result is declared. If it is negative, the quarantine is lifted, but if it's positive the home remains quarantined.

S4.1 Fig shows the effect of these different strategies. As shown in the main text, the benefit of the PCR tests' sensitivity is offset by the introduction of the delay, and using only PCR tests is no longer favourable. However, this can be countered by quarantining individuals or homes when the samples are taken. Quarantining homes still makes a larger dent in the total fraction of infected, however when large fractions of RAT tests are used (RAT:PCR $\sim$ 80:20) equivalent or marginally better results can be obtained by quarantining the homes of the tested individual when they are declared positive, instead of isolating them when they are sampled.

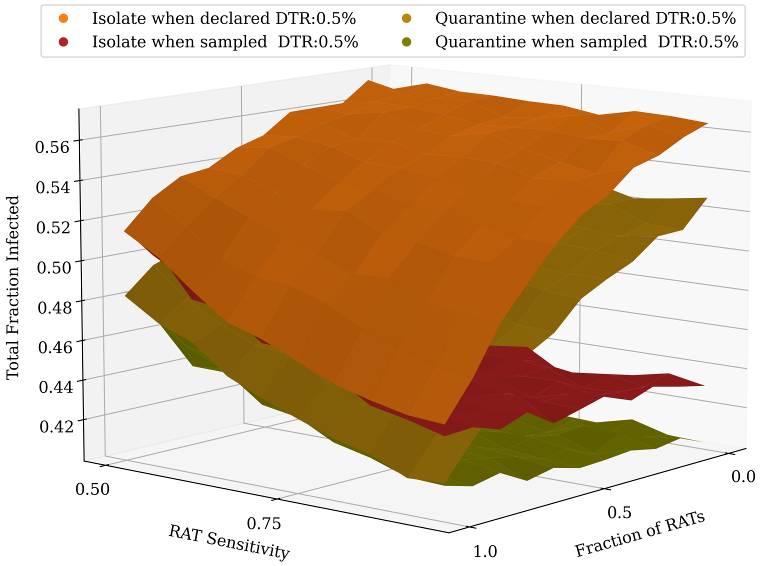


**(a)** Comparing all quarantining strategies for DTR 0.5%

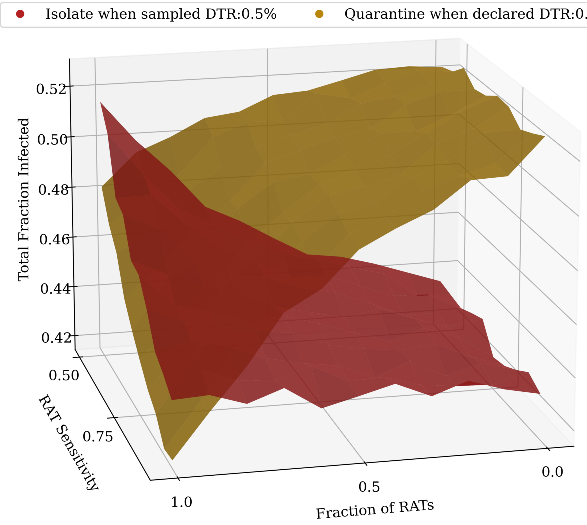


**(b)** Comparing isolating individuals when sampled with quarantining homes when declared

**S4.1 Fig: Comparing quarantining strategies with test delays.** Four different quarantining procedures are shown for a daily testing rate of 0.5%. Testing begins when 20% of the population has recovered. PCR tests have a 5 day delay, while RAT remain point-of-care tests. (a) The benefit of having more PCR tests in the mixture can be regained if the interventions are enforced when the test sample is taken. This effect is more significant at higher testing rates, though the trends remain the same. (b) At RAT:PCR ratios of 80:20, quarantining homes of those with positive results can do as well or even better than isolating all test candidates when they are sampled.
