# Supplementary material for: Optimizing testing for COVID-19 in India
Source: PLoS Comput Biol. 2021 Jul 22;17(7):e1009126. doi: 10.1371/journal.pcbi.1009126 (PMC8297905; doi:10.1371/journal.pcbi.1009126)
Supplement: S5 Appendix — Strategies that involve interventions being enforced when the individual is sampled are found to require a larger number of people and homes confined. We consider the case when only PCR tests are used with a delay of 5 days between sampling and the results being declared, since this leads to the largest number of people and homes quarantined. In the case of interventions that are enforced when the individual is sampled for a test, the number of people or homes confined does not die out even after the pandemic has passed, since people continue to be tested and remain confined for a duration of 5 days until the PCR result is declared. The peak number of people confined varies from 4% to 6%, and this translates into a fraction of homes quarantined between 15% and 25%. (PDF) [file pcbi.1009126.s005.pdf]

S5 Appendix: Cost of imposing interventions when test sample is taken

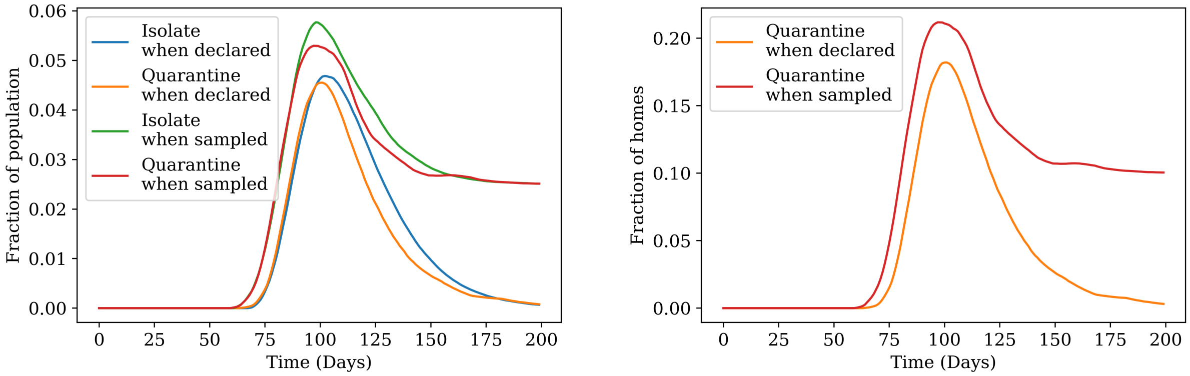

(a) Comparing number of individuals isolated **(b)** Comparing number of homes quarantined

S5.1 Fig: People and homes confined as a function of time. The plot shows the number of people confined on any given day given different quarantining strategies. The tests are assumed to all be PCR, and testing starts when 20% of the population has recovered. (a) Enforcing the intervention when the individuals are sampled leads to a higher peak, however the peak remains between 4% to 6% of the population. (b) This translates to around 15% to 25% of homes quarantined.

S5.1 Fig shows the total cost in terms of confining individuals and homes as a function of time, given different quarantining strategies. Strategies that involve interventions being enforced when the individual is sampled are found to require a larger number of people and homes confined. We consider the case when only PCR tests are used with a delay of 5 days between sampling and the results being declared, since this leads to the largest number of people and homes quarantined. In the case of interventions that are enforced when the individual is sampled for a test, the number of people or homes confined does not die out even after the pandemic has passed, since people continue to be tested and remain confined for a duration of 5 days until the PCR result is declared. The peak number of people confined varies from 4% to 6%, and this translates into a fraction of homes quarantined between 15% and 25%.
