# Supplementary material for: Optimizing testing for COVID-19 in India
Source: PLoS Comput Biol. 2021 Jul 22;17(7):e1009126. doi: 10.1371/journal.pcbi.1009126 (PMC8297905; doi:10.1371/journal.pcbi.1009126)
Supplement: S6 Appendix — As discussed in the main text, the majority of our simulations were carried out for the case where testing is begun when 20% of the population has recovered from the disease. As expected, the earlier one starts testing, the lower the testing rate needed for the same reduction in total infections. However, the relative impact of the different testing and quarantining strategies is similar, although quarantining of homes has a relatively more pronounced effect when starting at 5% seroprevalence. (PDF) [file pcbi.1009126.s006.pdf]

S6 Appendix: Effects of starting testing earlier

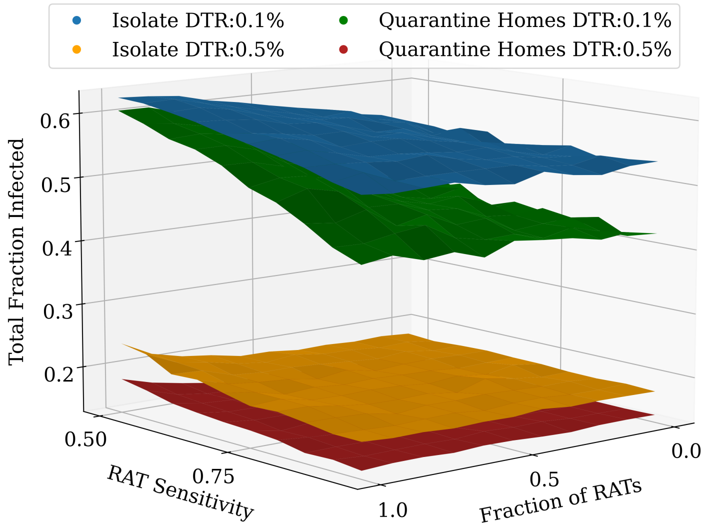

(a) Start testing when 5% have recovered

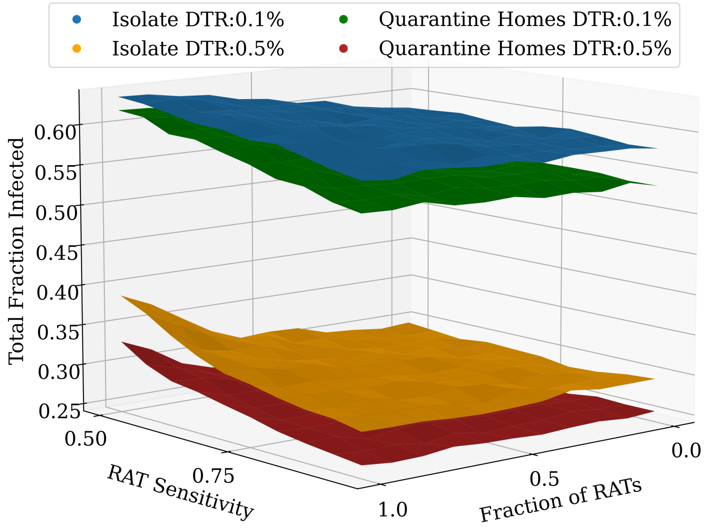

(b) Start testing when 10% have recovered

S6.1 Fig: Starting testing at lower seroprevalence The different testing and quarantining strategies were explored at different values of the background seroprevalence. As one would expect, the total number of recovered at the end of the infection is lower when we begin testing and quarantining earlier on in the infection. However, certain qualitative trends remain similar to Fig 8 in the main text: larger daily testing rates are seen to bring down the cumulative number of people infected by the disease, and quarantining the homes of those who test positive was found to be a better strategy than simply isolating the individual in their house. However, unlike the case when testing started at 20% of the population having recovered, the relative benefit of quarantining homes at low testing rates is much more significant. Apart from this, there is as before a general benefit of having a larger number of PCR tests in the test-mixture when RAT tests are of low-sensitivity, but this becomes less significant as the RAT sensitivity is increased.
