# Supplementary material for: Optimizing testing for COVID-19 in India
Source: PLoS Comput Biol. 2021 Jul 22;17(7):e1009126. doi: 10.1371/journal.pcbi.1009126 (PMC8297905; doi:10.1371/journal.pcbi.1009126)
Supplement: S7 Appendix — As shown in the main text, there exists a synergy between testing and masking, in excess of a simply additive or even multiplicative effect (see below). In the figure, we compare the effect that masking would have had if the effect had been purely additive or multiplicative. For a given daily testing rate of r, let f(r) describe the effect of testing (without masking), and g(r) describe the effect of testing and masking combined. We define ΔN = f(0) − g(0) to be the effect of purely masking the population. Then, by an additive effect we mean that: hadd(r)=f(r)-ΔN, and by a multiplicative effect we mean that hmult(r)=f(r)×(g(0)f(0)). All four functions f(r), g(r), hadd(r), and hmult(r) are shown in the figure. (PDF) [file pcbi.1009126.s007.pdf]

S7 Appendix: Synergy between testing and non-pharmaceutical interventions

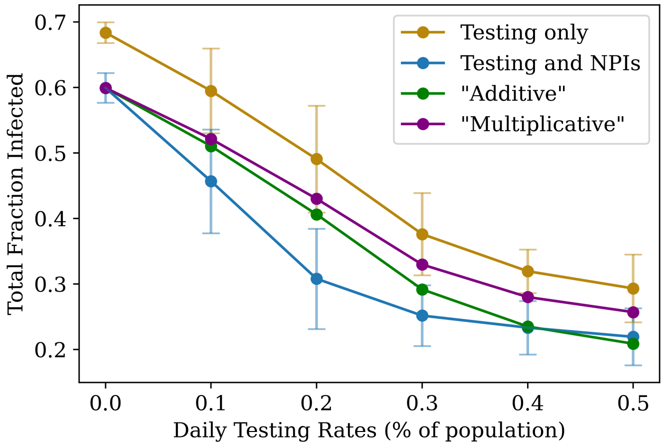

S7.1 Fig: Demonstrating the synergy between testing and NPIs. The effects of NPIs such as masking on testing is found to be lower than if it had been purely additive or multiplicative.

As shown in the main text, there exists a synergy between testing and NPIs. In S7.1 Fig, we compare the effect that NPIs such as masking would have had if the effect had been purely additive or multiplicative. For a given daily testing rate of r, let the function $f(r)$ describe the effect of testing (without NPIs), and $g(r)$ describe the effect of testing and NPIs combined. We further define $\Delta N = f(0) - g(0)$ to be the effect of the population purely using NPIs. Then, by an additive effect we mean that:

$$h_{\text{add}}(r) = f(r) - \Delta N,$$

and by a multiplicative effect we mean that

$$h_{\text{mult}}(r) = f(r) \times \left(\frac{g(0)}{f(0)} \right).$$

All four functions $f(r), g(r), h_{\text{add}}(r)$, and $h_{\text{mult}}(r)$ are shown in S7.1 Fig.
