# Supplementary material for: Optimizing testing for COVID-19 in India
Source: PLoS Comput Biol. 2021 Jul 22;17(7):e1009126. doi: 10.1371/journal.pcbi.1009126 (PMC8297905; doi:10.1371/journal.pcbi.1009126)
Supplement: S8 Appendix — (PDF) [file pcbi.1009126.s008.pdf]

S8 Appendix: Flowcharts of Simulation

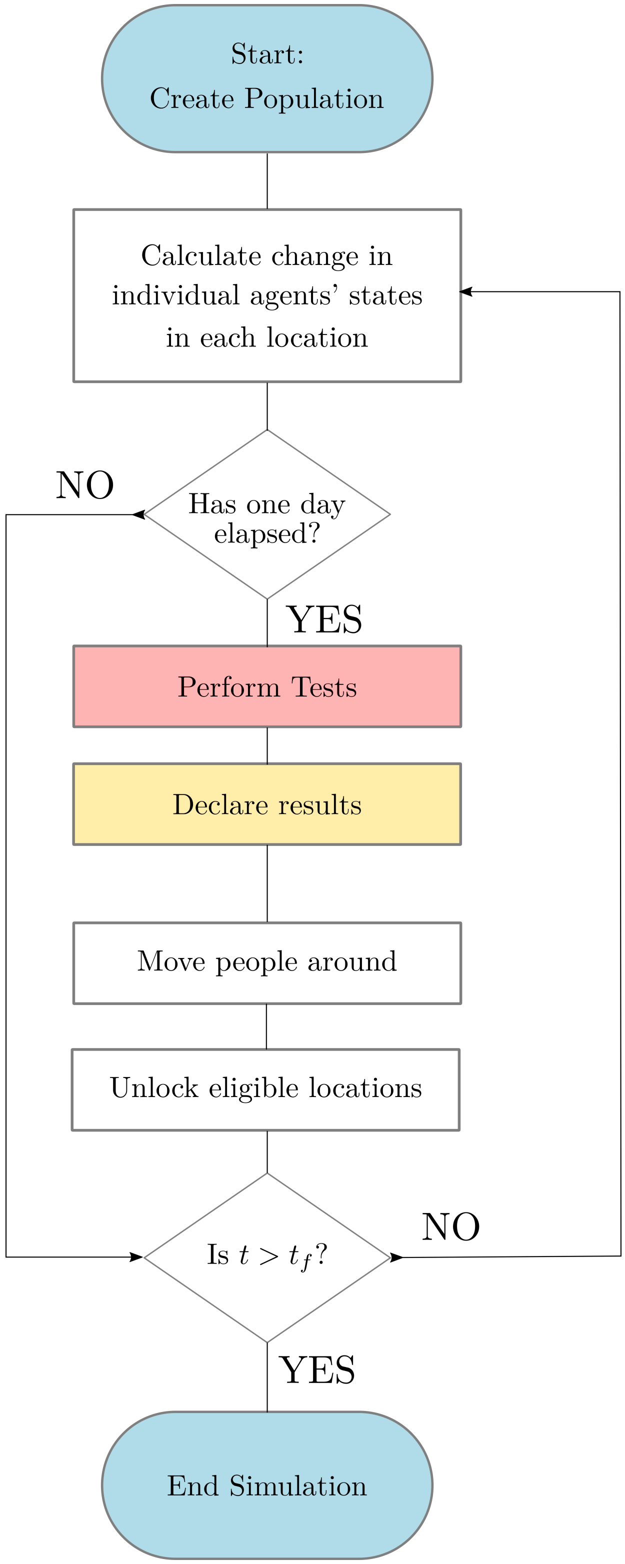

S8.1 Fig: Global schematic of the simulation.

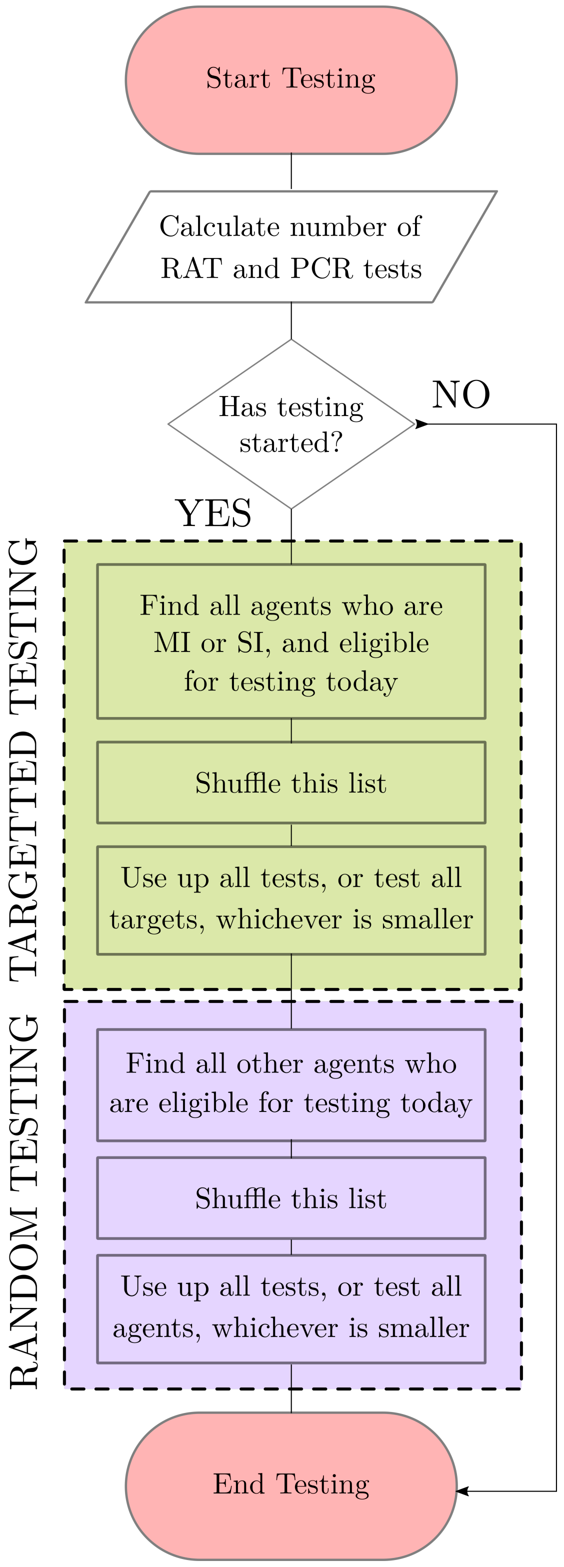

S8.2 Fig: Schematic of testing individuals.

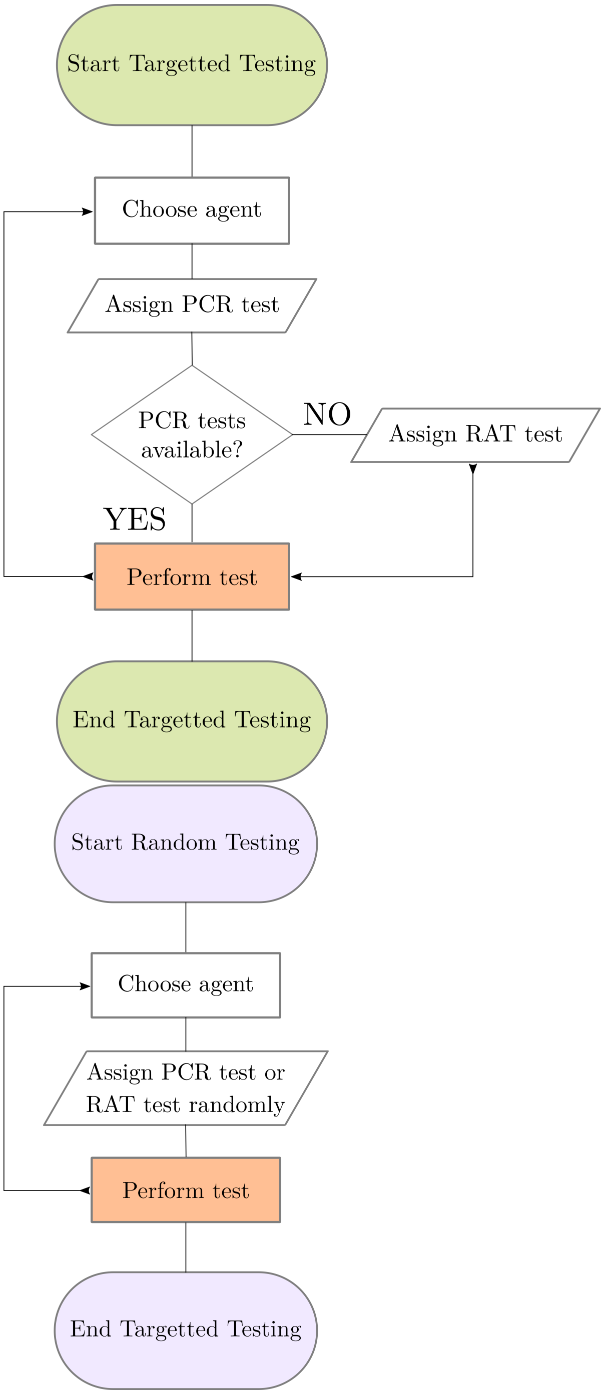

(a) Schematic of targeted and random testing.

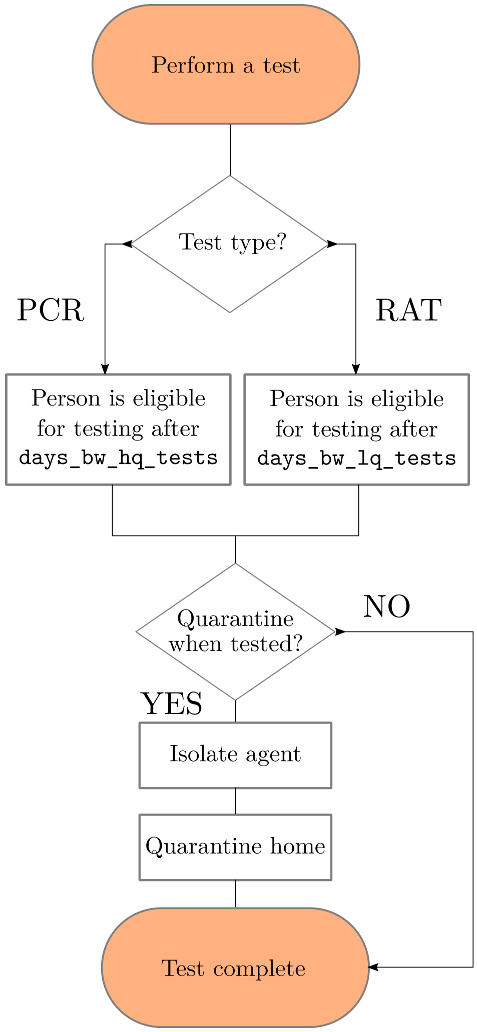

(b) Schematic of performing a test.

S8.3 Fig: Schematic of testing procedures.

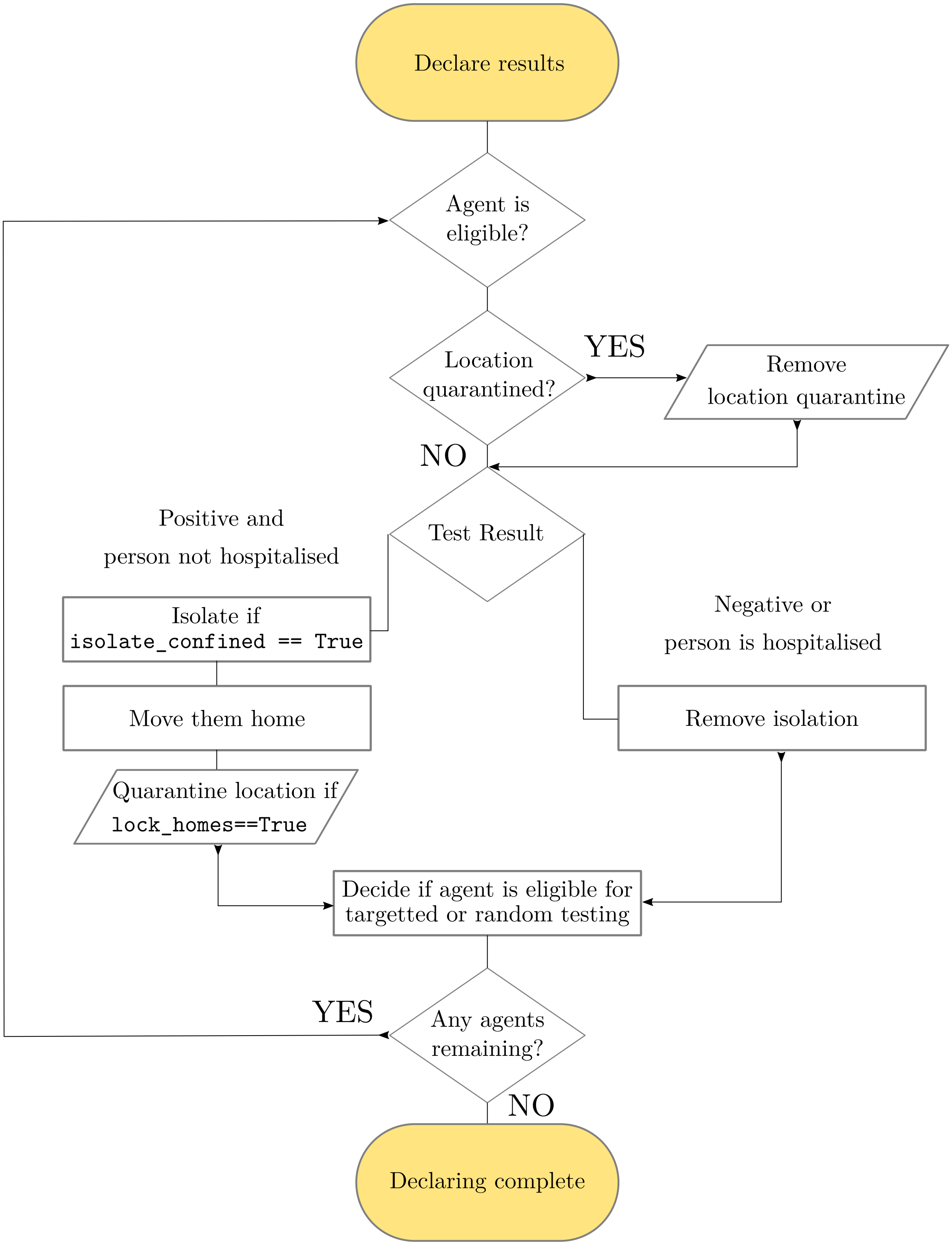

S8.4 Fig: Schematic of declaring results.
