# Supplementary material for: Optimizing testing for COVID-19 in India
Source: PLoS Comput Biol. 2021 Jul 22;17(7):e1009126. doi: 10.1371/journal.pcbi.1009126 (PMC8297905; doi:10.1371/journal.pcbi.1009126)
Supplement: Attachment — Submitted filename: Responses_to_reviewers.pdf [file pcbi.1009126.s009.pdf]

Responses to Reviewers

Philip Cherian, Sandeep Krishna, Gautam I. Menon

May 12, 2021

We thank both reviewers for their careful reading and incisive comments. We have addressed all the points raised by the reviewers in the revised manuscript, as detailed point by point below. We have also made some minor modifications to the text to mention changes in the pandemic situation since the time of our first submission. Our replies below, and all changes to the revised manuscript are marked in blue colour:

Reviewer #1: The authors have developed a network-based model to assess different diagnostic approaches to COVID-19 in India. This is an important question to explore through mathematical modeling and relevant to all geographies – India and elsewhere. Diagnostics are one of the three primary interventions that can help reduce the morbidity and mortality associated with COVID-19. The manuscript is very clearly and concisely written. The methods are very clearly described and seem perfectly appropriate to address the question raised by the authors. I have one overarching comment and several minor comments. Otherwise, this is an important manuscript and will contribute to ongoing efforts to address the epidemic in India and elsewhere.

MAJOR COMMENT

- The authors have assumed 100% sensitivity for RT-PCR and varying levels of sensitivity for rapid antigen tests. The discussion/conclusions could be strengthened by acknowledging recent evidence that brings this assumption into question. In a study published by Pekosz, et al. in Clinical Infectious Diseases (CID) indicates that rapid antigen testing correlates with culturable virus, indicating that viral particles can be transmitted when someone tests positive by such tests; whereas RT-PCR does not correlate with culturable virus. The positive predictive value of antigen tests was considerably higher than RT-PCR. This has been attributable to the sensitivity for viral RNA rather than detection of surface protein. This could give rapid antigen tests and even greater advantage over RT-PCR. I think it would be difficult for the authors to incorporate this point in their existing model; however, a recognition of this point and the directionality of the potential bias introduced into their model mentioned somewhere in the manuscript could be helpful for the reader.

  We agree with the reviewer. In fact, both points strengthen our conclusion that using rapid, low sensitivity tests can provide similar (or better) epidemiological outcomes. We have added a paragraph to discuss this in the Conclusions section (lines 430-439).

- In addition, I am aware that in many settings where rapid antigen tests have been used, a testing algorithm has been employed. So, for example, for a SARI or ILI patient, a rapid antigen test is first used. If the patient is positive, then they are considered positive. However, if a test is negative, given they are then tested by RT-PCR. Is this happening at all in India?

Another interesting paper found that a commonly used rapid antigen test found that using two tests consecutively increased the sensitivity to 99%. Slovakia, admittedly with only 5.4 million people, tried to test the entire country at once. Might the authors be able to look into comparing such testing algorithms? Or at least mentioning them as additional points to explore in future research?

Indeed, in some areas in India for a period of time a negative RAT test was followed by a PCR test. We had not made it clear enough in the manuscript that we implement a version of this in our algorithm for targeted testing of symptomatics: if a person who is symptomatic had a negative RAT test at least 7 days earlier, then they are prioritized in our target list and are therefore more likely to receive a PCR test. We found that this prioritization does not make much difference to the epidemic spread. We have modified lines 197 and 198 in the Methods section to make this more clear to readers. Additionally, we have commented on the effect of using two consecutive RAT tests to raise the sensitivity to 99% in the Conclusion section (lines 430-439). Given our results, our prediction for such a use of RATs is that the gain in sensitivity would not overcome the loss of coverage due to there being fewer effective tests per person.

MINOR COMMENTS

(a) **Introduction**

- The authors cite a Wikipedia article when stating that that the stringently enforced lockdown in India contributed to slow epidemic growth. Surely there must be more appropriate references for this.

  We have rewritten the first paragraph of the introduction to simply provide the numbers of cases (from the database `api.covid19india.org`) at various points, such as the end of the lockdown and the following months. One can see that increase of cases did accelerate once the lockdown ended, but we leave it to the reader to come to their own conclusions about whether this is causally related.

- The final paragraph of the introduction beginning on line 93 provides an interpretation of the findings. I will defer to the editors regarding whether this is status quo for this journal. It would seem more appropriate as part of the discussion section. However, if the editors indicate that it should remain in the introduction, it would be important to include some of the limitations of the model findings in this paragraph.

  We have removed this paragraph. All the points mentioned here are in fact already mention in the Conclusions, so we did not feel there was any point moving it to the Conclusions section.

(b) **Model and methods**

- In table 1, it would be helpful for readers if the authors could provide the epidemiologic definitions for each of the model parameters.

  We have added this to the Table

- Beginning on like 167, the authors note that health care workers are 25-30% more likely to become infected compared with non-health care workers in the absence of testing. Is this based on the observations made from the model? Or from empiric evidence? If the

latter, more details would be helpful. If from the former, I would suggest including in the results rather than the methods.

This was indeed not clearly explained in the manuscript. This is in fact not a result, because we used the 25-30% number to choose the value of the parameters that describe the reduction in transmission for patients who are hospitalized. We have altered lines 168-171 in the Methods section in order to make this more clear and to cite the empirical evidence that suggests the 25-30% number.

(c) **Models for testing and quarantining**

- The authors start testing after 20% of the population has been recovered. What is the justification for this? Testing in India has scaled up relatively fast and test positivity in most states remained below the 5% target likely before the 20% seropositivity. Different starting times are then presented in Figure 4. In reality, testing would be scaled up over time rather than once a certain threshold is attained. Can the authors comment on this?

  Our main aim was to investigate the role of low sensitivity tests and different quarantining strategies during intermediate levels of seroprevalence. At the time of writing 20% was the seroprevalence in several parts of India, in particular UP and Bihar, and in this context there was a vigorous debate about whether to use RAT or PCR tests, which we hoped to contribute to. In fact, many of our qualitative results hold even when testing starts at a lower seroprevalence. We have now included in Supplementary material heatmaps, similar to Fig 8 in the main text, for testing starting at 5% and 10% seroprevalence. In our simulations, the seroprevalence at the peak of active infections is around 22-23%, hence we have not explored higher starting seroprevalences because that's when the epidemic is already on its way down. This is discussed in a new paragraph in the Conclusions section - lines 459-473. We also comment there on what we might expect if testing ramped up slowly in time rather than as a step function.

- For eligibility of testing, did the authors consider asymptomatic individuals identified through contact tracing? If not, what are the implications of not including these individuals in the model?

  The reviewer raises a very interesting point. We in fact tried one simple contact tracing algorithm, where we determined the primary contacts of a positive individual for the 14 days prior to them being declared positive, and added these people to the priority list in our targeted testing. It turned out that this made little dent to the epidemic, because at the testing rates we explored the number of contacts was far larger than the number of tests available each day and too many tests were being wasted on people who were contacts but were not actually infected. In our simulations, when there are limited tests per day, it seems simpler and better to target symptomatic individuals irrespective of their contact history. However, there might well be more sophisticated contact tracing algorithms one could usefully combine with such testing that is targeted at symptomatics. We have added a paragraph in the Conclusions section, lines 501-511, to discuss this issue.

- Beginning on line 196, the authors note that unused tests are distributed randomly among eligible individuals in the population. Why was this approach adopted? Surely, some of the states in India have used tests in random testing. But I would imagine this would be done using RAT rather than RT PCR. Can the authors expand on this

approach?

We had not explained our approach clearly in the manuscript and we thank the reviewer for pointing this out. We have now tried to make our targeted testing algorithm clearer by rewriting parts of the "Eligibility for testing" section in Methods. Given a certain number of tests available per day, some of which are PCR and some RAT, we first use PCR tests on symptomatic individuals who are eligible for testing (some symptomatics are excluded, e.g. those who have already tested positive, or those who are hospitalized, etc.). Then, we use RAT tests on any remaining eligible symptomatic individuals. At any point if there are no remaining symptomatic individuals who are eligible for testing, only then do we use any remaining tests for random testing. Thus, during the peak of the infection - typically from the time when tests start to to 20-40 days later - the number of tests administered to symptomatic individuals dominates the number administered to randomly chosen (non-symptomatic) individuals. By the time the number of random tests starts to dominate, the epidemic is typically past the peak and on its way down. Further, because we use PCR tests first and only then RAT tests, when the tests start being given randomly they are mostly RAT tests. We have rewritten parts of the section "Eligibility for testing" in an attempt to make this more clear.

Reviewer #2: This paper aims to model the consequences of using antigen vs PCR tests for COVID surveillance testing, specifically in the context of India. The paper assesses the consequences of varying the fraction of tests which are antigen vs PCR, as a function of the sensitivity of the antigen test. It is found that when the antigen test is at least moderately sensitive, incorporating a high fraction of antigen tests into the surveillance testing program is effective at reducing infections.

Assessing these specific tradeoffs in the design of surveillance testing programs is a timely and worthwhile goal, as is exploring them specifically in the context of India. Overall, the results seem fairly sensible. Some specific comments about individual parts of the paper:

- The model description is very unclear and hard to follow. First, a compartmental ODE model is presented. Then, an individual-level network simulation is introduced. What is the relationship between the ODE formulation and the individual-level, discrete-time network model? And, how is the force of infection (Eq. 9) formally used in the network model? If the network model is what is used throughout the paper, a complete formal description of it should be given somewhere in the paper.

  Indeed, we only use the network model, so we have removed the description of the compartmental from the main text and placed it in the supplement. The compartmental model arises as a special case of the network model if there was only one single location where all individuals remained. We have also rewritten the section in Methods on the network model in various places (e.g., sections "network model description" and "network model implementation") to make clear how the disease stochastically progresses within individuals, how the expression for force of infection is used, and how individuals move from one location to another. These are the only rules required to fully specify the network model dynamics in the absence of testing. The section on testing then describes how testing and quarantining rules are added to this. We hope this organization makes the model much clearer.

- A sensitivity analysis would be helpful to test the impact of delays in reporting of PCR test results. It is unsurprising that 5 days is too long for the results to be useful, but what about an intermediate length of time?

We did such a sensitivity analysis by examining PCR tests with delay 1 and 2 days. We found a delay of 2 days for PCR tests was sufficient to offset the benefits of a perfect PCR test, that is, with a delay of 2 days all test mixtures were just as effective, irrespective of the sensitivity or proportion of RATs in the mixture. In such cases, increasing the overall daily testing rate or implementing a different quarantining strategy was found to give much better results than increasing the ratio of PCR to RAT tests.

- The model seems to assume that both antigen and PCR tests have uniform sensitivity over the course of an individual's infection? This is not consistent with available evidence. Of course some simplifying assumptions are reasonable, but some basis (e.g., sensitivity analysis) should be given for thinking that this does not impact the results too much.

  The reviewer is correct that we assume the sensitivity is uniform over the course of an individual's infection. In reality, the viral shedding by individuals will likely start low, then increase with time and later decrease over the course of their infection. The main effect of including this would be to increase the false negatives for both RAT and PCR tests, i.e. to decrease their effective sensitivity. This would not change any of our conclusions. Any additional correlations associated with the sensitivity being lower at specific times in an individuals' infection would likely be wiped out by the many sources of stochasticity in our simulations which prevent synchronicity of new infections.

- How many simulations do the results average over? It seems like some of the variation, particularly in the left-hand plot of Figure 6, might be due to random variation between runs.

  All averages are over 100 simulations. We have now mentioned this in every Figure caption. Indeed the variation is due to random variation between runs. The figures with ellipses (Fig 5 and 10) depict this variation - each point there corresponds to a single run, so the extent of each ellipse depicts the variability from run to run.

- What is the rationale for including quarantine strategy C? It seems very difficult to motivate a policy which preemptively quarantines people who are randomly selected for surveillance testing, i.e, are asymptomatic and have no known exposures. I would recommend just removing this scenario.

  We used this strategy to show the reader an extreme limit. We have now acknowledged in lines 231-235 that this would be unrealistic to implement practically once the epidemic is past the peak and most of the tests are randomly administered. However, initially, for 20-40 days after testing starts, the tests are mostly administered to symptomatic individuals, so in this scenario it might be possible to implement immediate quarantining. Indeed, in India self-quarantining has been advised to individuals who have symptoms but have not received a test or are waiting for a result, though the compliance rate is not known.

- The description of Figure 13 as studying "masking" is a little strange since it is implemented as a generic reduction in the infectivity of the disease – is this distinguishable in the model from the effect of any generic NPI, e.g. social distancing? I would describe this just as measuring the interaction between testing and any combination of other interventions which reduce beta.

  We agree with the reviewer and have changed all references to masking to refer to NPIs instead.

- In the discussion, some of the results are intuitively motivated by asking what level of testing would allow all new cases to be located (line 372). I don't think that this is the right intuition. To drive cases to 0, we just need $R < 1$, not that every new case can be found. Also, the modeled policies are allocating many/most of their tests to random surveillance testing, which isn't targeted to exactly finding the newly infected people.

  We thank the reviewer for pointing this out and we agree - indeed this intuition provides an upper bound on the number of new cases that need to be caught each day in order to dampen the epidemic, and in reality one only needs to drive $R < 1$. However, we nevertheless find that this intuition is useful for two reasons: (i) this bound is easier to estimate and think about, especially because it also refers to an instant in time whereas the reproduction number refers to the number a person would infect over an extended period of time, which is harder to estimate while a pandemic is ongoing, (ii) despite being only an upper bound it nevertheless matches reasonably well with the daily testing rate at which one starts to see diminishing returns. This is because our testing algorithm is targeted somewhat towards finding new cases - since it is targeted at symptomatic individuals and these remain dominant over random testing for 20-40 days after testing starts. Thus, we have retained our discussion of this intuition but we have also added the reviewer's point about the real need being to bring $R < 1$ in lines 396-399 and mentioned that this intuition only provides an upper bound on the number of new cases that need to be caught.

- How long is the simulation run for? Figure 6 just claims to report the fraction infected after "some time".

  Thank you for pointing out this vague statement. All simulations we run for 200 days. We have now included this in all figure captions.
